# Ascorbic Acid for the Safe Use of a Sunscreen Agent: Accumulation of Nano Zinc Oxide and Titanium Dioxide on the Skin

**DOI:** 10.3797/scipharm.1306-05

**Published:** 2013-07-14

**Authors:** Anahita Fathi-Azarbayjani, Poh Leng Tan, Yew Ying Chan, Sui Yung Chan

**Affiliations:** 1 Department of Pharmacy, National University of Singapore, Block S4, level 2, Science Drive 4, 117543, Singapore.; 2 Department of Medicine, Urmia University of Medical Sciences, Urmia, 5756115111, Iran.; 3 Department of Anesthesiology, Singapore General Hospital, 169608, Singapore.

**Keywords:** Zinc oxide, Titanium dioxide, Ascorbic acid, Penetration enhancer, Skin permeation

## Abstract

**Objective:**

Physical UV absorbers such as titanium dioxide or zinc oxide have been found to be highly protective against ultraviolet radiation. Sun protection factor depends on the accumulation of the minerals on the skin. UV-absorbing agents must accumulate within the upper skin layers in order to provide a dense light-absorbing layer and guarantee water resistance. The aim of this work was to increase the skin deposition and efficacy of sunscreens without increasing their skin permeation. The application possibility of EDX to determine the quantitative elemental composition of zinc and titanium on the skin surface was studied.

**Method:**

The changes induced in the skin deposition of physical UV absorbers in conjunction with ascorbic acid were studied. *In vitro* skin permeation and X-ray elemental analysis were carried out to determine the mineral skin deposition effect of ascorbic acid.

**Key findings:**

Results indicate that ascorbic acid may significantly increase the skin deposition (p < 0.05) of these minerals on the skin without increasing their skin permeation (p > 0.05). Flow through diffusion cell and X-ray elemental analyses appear to be complementary and show that ascorbic acid is able to increase accumulation of sunscreen on the skin.

## Introduction

Physical sun blocking agents such as titanium dioxide and zinc oxide have been found to be highly protective against ultraviolet, visible, and even infrared radiation. They are photostable: they do not react with organic sunscreens. However, these mineral sunscreens tend to be white or opaque on the skin and therefore cosmetically unacceptable. This can be overcome by micronisation to decrease particle size and produce particles that are transparent to visible light and appear natural and invisible on the skin [1, 2]. Based on previous studies, there is no evidence of significant penetration of titanium dioxide and zinc oxide particles beyond the stratum corneum. The extent to which topically applied mineral sunscreens can penetrate the skin has been studied extensively and results suggest that the skin penetration of these particles is negligible [3, 4].

*In vitro* studies have found no evidence of significant penetration of titanium dioxide and zinc oxide nanoparticles beyond the stratum corneum [5], and further *in vivo* studies on human subjects with UV exposure revealed trace amounts of Zn in blood and urine after a 5-day application phase which indicates low levels of absorption through the skin [6]. Previous studies demonstrated that topically applied nano TiO_2_ particles did not penetrate beyond the stratum corneum and TiO_2_ aggregates were localized on the top of the human epidermis [7, 8].

UV-absorbing agents must accumulate within the upper skin layers in order to provide a dense light-absorbing layer and guarantee water resistance. One method to solve this poor water resistance is to develop sunscreens with high affinity to the skin layer to increase the retention on the upper skin layers for a certain period of time [9].

Topical antioxidants diminish UV-related damage of theskin. They scavenge and destroy free radicals and oxidizing agents. Therefore, a variety of marketing strategies use these antioxidants, ascorbic acid, tocopherol, and beta-carotene, in combination with sun block [10].

Scanning Electron Microscopy coupled with Energy Dispersive X-ray Spectroscopy (SEM/EDX) is a surface sensitive technique for qualitative evaluation of elements. The X-ray intensity is proportional to the number of elemental atoms and their weight in the sample. The technique is multi-elemental and the detection limit is about 1000 ppm and can be reduced by using long counting times. The X-ray lines produced are representative of the shell (K, L, or M) containing the inner vacancy of the element, for example Zn-K and Zn-L refer to the K and L shell of zinc [11].

The aim of this work was to study the application possibility of EDX to determine the quantitative elemental composition of zinc and titanium on the skin surface. The purpose was to explore if ascorbic acid may enhance skin accumulation and topical deposition of physical UV absorbers to enhance the sunscreen efficacy. Minerals’ skin deposition was determined using X-ray elemental analysis, and *in vitro* skin permeation studies were carried out using a human epidermis and flow through a diffusion cell. Morphologies of the nanoparticles and the treated skin samples were observed.

## Methods

### Materials and Methods

L-ascorbic acid, zinc oxide, and titanium dioxide nanoparticles (<100 nm), were purchased from Sigma, Singapore. All other ingredients used were of analytical grade.

### Preparation of Formulations

An oil-in-water emulsion consisting oleic acid 1: propylene glycol 1: Tween 20 1: deionized water 7, were used as the vehicle. Details of the formulations are illustrated in Table 1.

### Field Emission Scanning Electron Microscopy

The appearances of zinc oxide and titanium dioxide nanoparticles and the electron mapping were examined using a field emission scanning electron microscope (Jeol JSM-6701F; Japan) operating at an accelerating voltage of 5 kV equipped with an energy dispersive X-ray force. Samples were coated by a sputtering unit (Jeol JFC-1600 auto fine coater, Japan).

### UV Absorption Studies

Qualitative UV absorbance spectra from the formulations were obtained using a Perkin Spectrophotometer (Spectrum 100 USA) in a wavelength range between 280 and 380 nm.

### Skin Permeation Studies

Skin samples of healthy adult Chinese females were obtained with patient consent and ethics approval, after abdominal reduction surgery. This study was approved by the Institutional Review Board (IRB) of Singapore General Hospital, Republic of Singapore (IRB Reference Number 196/2006). This IRB operates in accordance with the International Conference on Harmonization/Singapore Guideline for Good Clinical Practices, and with the applicable regulatory requirements. The well-established and commonly used heat separation method was employed for the separation of epidermis. Subcutaneous fat was carefully separated from the epidermis after immersing the whole skin in distilled water at 60 ± 5°C for 2 min. Samples were stored at −80°C until use. Prior to the permeation studies, the skin samples with the stratum corneum side facing upwards, were equilibrated for 2 h in 0.9% w/w sodium chloride solutions containing 1% v/v antibacterial antimycotic solution. This heat treatment method has been widely used as the preferred separation method for percutaneous absorption experiments [12].

Permeation studies of sunscreen emulsions were performed using a flow-through diffusion cell apparatus. The donor compartment was filled with 1 ml of the topical formulation and the receptor compartment was a phosphate buffer and the pH was adjusted to 5. The exposed surface area of the skin samples for the permeation studies was 0.785 cm^2^. Cell temperature was kept at 37 ± 0.5°C throughout the experiment. After a 24-h period, the skin samples were analyzed by X-ray spectroscopy to quantify the skin mineral deposition. Experiments were carried out in triplicates.

### Skin Histology

Skin samples used in the diffusion studies were processed for light microscopy. Samples were soaked overnight in 85 ml of 80% v/v ethanol, 10 ml formaldehyde, and 5 ml acetic acid (Gasper et al. 2007). After a series of dehydration, they were embedded in paraffin and semi-thin sections were stained with hematoxylin and eosin prior to being examined with a light microscope (Leica EC 3, USA).

### Energy Dispersive X-Ray Spectroscopy (EDX) Analysis and Skin Deposition Studies

EDX measurements were carried out by means of a FESEM equipped with an energy dispersive X-ray source to identify and quantify the zinc and titanium that remained on the skin after permeation. Skin samples collected after skin permeation studies were thoroughly washed with phosphate buffer saline (PBS). After coating with platinum, samples were analyzed at 15 kv voltage. The area to be analyzed was selected and the electron beam scanned the skin area and identified the intensity of the characteristic X-ray energies of specific elements. The X-ray spectra provided data on the various elements, as well as their relative percentages present on the skin.

X-ray counts from thousands of points on the skin surface were collected in 30 min and the elemental map was obtained. The X-ray peaks generated at the end of the scanning time correspond to the weight of the identified element that existed on the skin. K lines were used in each scan for zinc and titanium. For each analysis, a minimum of five measurements were conducted. Mean and standard deviation (SD) of each element concentration were calculated from the repeat analysis. The untreated skin sample was used as a control to correct the background peaks for zinc and titanium.

### Statistical Analysis

Results were expressed as the mean ± S.D. of at least three experiments. Analysis of variance (ANOVA) was carried out (Graph Pad Prism, Version 5.0) followed by the Tukey post-hoc test to determine the differences between treatment groups. A value of p < 0.05 was considered statistically significant.

## Results

### Field Emission Scanning Electron Microscopy (FESEM)

**Fig. 1:**
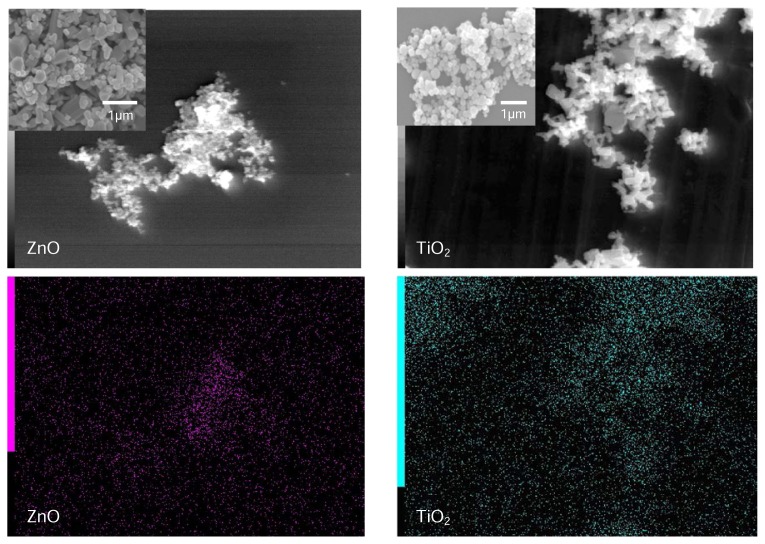
Particle morphology and mapping of znO and TiO_2_ nanoparticles in the emulsion.

FESEM was used to view the shape, size, and distribution of nano-sized TiO_2_ and ZnO in formulations. It is evident that the particles have agglomerated in the emulsion. Zinc oxide consisted of rod-like (80nm nm in diameter and 250 nm in length) and spherical particles (with an average diameter of 90 nm). Titanium dioxide was mainly in a spherical shape with an average diameter of 80 nm (Fig. 1).

### UV Absorption Properties of Ascorbic Acid and Formulations

**Fig. 2:**
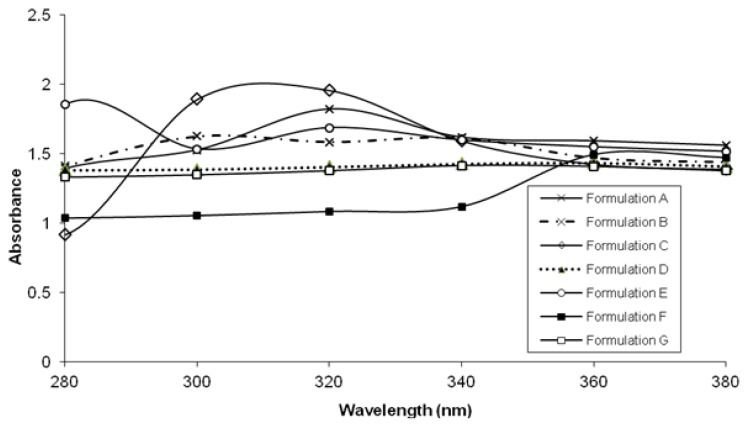
UV absorption of various formulations.

A UV absorption plot in a range of 280–380 nm of all formulations is shown in Fig. 2. It was observed that ZnO 5% presented a very high UV absorption along the scanned range having only one-centered peak located at 310 nm, whereas in ZnO 5% with ascorbic acid, two maximum peaks located in a range of 290 and 320 nm were detected. Samples with ascorbic acid had a slightly different pattern and lower absorption rate. The absorbances reported for TiO_2_ 5% suspensions were low, meanwhile those containing 5% ascorbic acid were remarkably increased. It could be speculated that different chemical complexes were established between ascorbic acid and nano ZnO/TiO_2_.

### Skin Permeation Studies

**Fig. 3:**
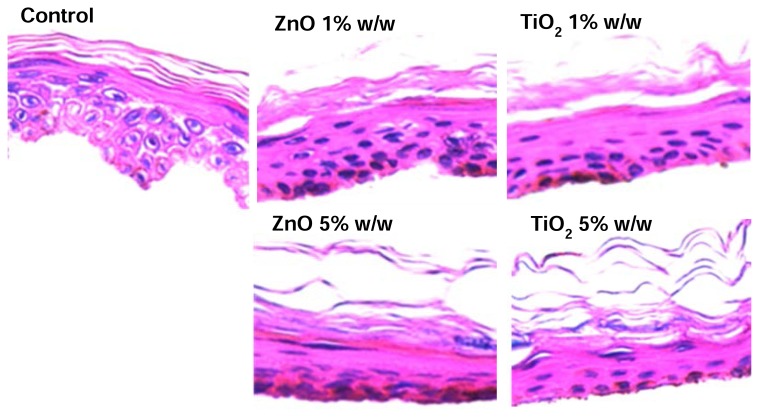
Morphology of human epidermis, histological skin examinations were performed *in vitro* 24 h after skin permeation studies, (×400). The nucleated cells of the epidermis have been stained blue, unsaturated lipids, including fatty acids and esters have been stained red.

The microscopic appearance of the skin treated with various minerals is shown in Fig. 3. In the control, a clearly defined SC could be seen. After treatment, minerals detached from the SC layer. The SC layer was fragmented and an enlargement of inter-keratinocyte spaces was observed. Evaluation by light microscopy showed dense structures of mineral agglomeration of the skin.

**Fig. 4:**
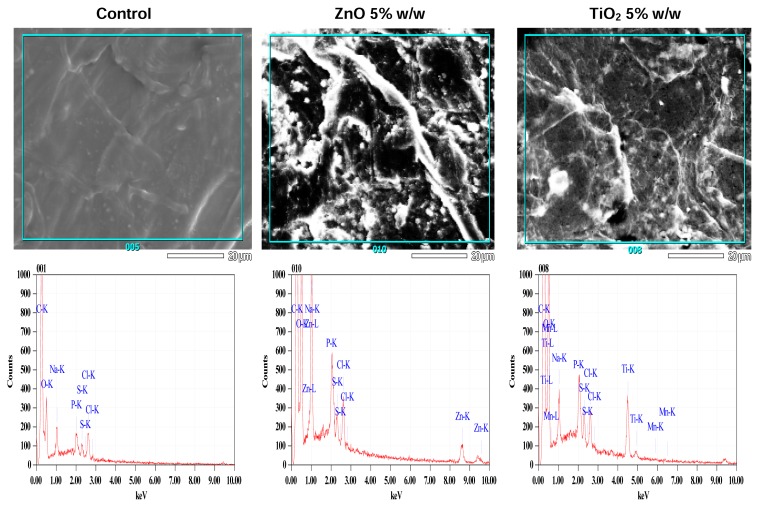
X-ray energy spectrum of human epidermis treated with formulations, demonstrating the presence of the mineral-specific signals.

Fig. 4 illustrates the mineral deposition on the skin surface studies by the EDX method. The FESEM study of treated skin revealed a flaky appearance of the corneocytes in the treated samples. The spectra of various elements present on the control skin surface and samples treated with nano zinc oxide and titanium dioxide formulations are shown in Fig. 4. The presence of Na, Cl, and P is due to the washing of skin samples with phosphate buffer saline (PBS). Manganese detected from the skin samples treated with titanium is due to doping, replacing some titanium dioxide with a small percentage of a transition metal, manganese (Mn). This enhances photostability and decreases the light-scattering properties of titanium. Each peak represents the mass percentage of the element present in the area and only the peaks from the k shell of the elements were detectable. Owing to the greater difference in the peak placement of Zn-k and Ti-k, it is easier to differentiate between the two signals and calculate the amount of elements present without interference and overlapping. The signal for Zn-L, Ti-L, and Na-K seem to overlap, however the energies from these shells were small and they were not used in the analysis.

**Fig. 5:**
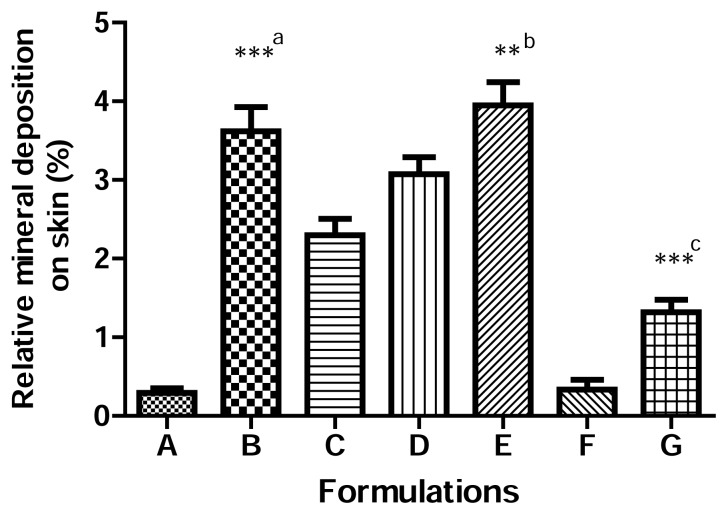
Mineral skin deposition after 24-hr application. ^a^ p<0.001 vs Formulation A, ^b^ p<0.01 vs Formulation C, ^c^ p<0.001 vs Formulation F.

Fig. 5 compares the percentage of skin surface deposition of the minerals after treatment. The total applied amount of zinc and titanium was recovered from formulations that did not contain ascorbic acid. These results are consistent with the evidence published in previous papers and show that a simple washing procedure is sufficient to remove minerals from the skin surface without any penetration [1, 3]. Hence, presence of ascorbic acid increased the deposition of zinc and titanium on the skin surface significantly (p < 0.05). However, there was no skin permeation observed and the amount of zinc and titanium found in the receptor fluid was comparable in formulations with or/without ascorbic acid. There was no significant difference in the skin permeation of nanoparticles from the test and control samples across human epidermis after 24-hour application of the minerals on the skin, p>0.05, results not shown. Also, light microscopy and FESEM confirm the presence of these nanoparticles on the skin surface. This suggests that ascorbic acid may increase the solubility of the minerals and enhance their deposition on the skin surface without increasing the permeation through skin layers. The more prominent effect of ascorbic acid on the skin deposition of nano zinc oxide is due to its higher water solubility as compared to nano titanium dioxide. The effect of ascorbic acid and its derivatives to increase drug solubility and enhance the concentration-dependent drug permeation across the skin has been studied [13, 14]. The FTIR spectra of ascorbic acid-treated rat epidermis showed concentration-dependent interaction with epidermis keratin, protein, and lipid which altered the barrier properties of the stratum corneum. It was speculated that owing to its low molecular weight, ascorbic acid (176 Da) can hydrate keratin and occupy hydrogen-binding sites on the protein structures in the corneocytes and interact with the polar heads of the intercellular lipids [15, 16].

It can be concluded that the interaction of ascorbic acid and the physical sunscreen formulations can enhance the efficacy and increase deposition of mineral sunscreens on the skin surface. Since users are often encouraged to liberally apply sunscreen before sun exposure, and to reapply every 2 hours, concomitant use of mineral sunscreens containing ascorbic acid or its derivatives could increase mineral deposition on the skin and reduce the need to reapply the sunscreen. The better accumulation of the pigments in the upper parts of the skin may increase water resistance and may lead to an increase in sun protection factor.

Further dermal risk assessment of nano zinc oxide and titanium dioxide in combination with ascorbic acid in sunscreens should be considered to determine if this conclusion holds under normal conditions of sunscreen use. There is a need to study the implications of this finding for cosmetic applications.

## Conclusion

EDS is a sophisticated analytical technology to identify metal present on the surface of the sample. Analysis of the receiver fluid of the skin permeation studies shows that these elements did not penetrate the epidermis layer and there was no zinc and titanium in the receiver compartment (minimum detection limit 5 μg/ml). These findings are consistent with previous *in vitro* and *in vivo* studies which suggest that the skin penetration of these mineral nanoparticles is negligible. This study shows that ascorbic acid is able to further increase accumulation of sunscreens on the skin surface and enhance their efficacy.

It can be concluded that nanoparticles of mineral UV absorbers appear safe and exhibit minimal skin permeation in ascorbic acid-containing formulations and their skin accumulation is enhanced when compared to formulations without ascorbic acid.

## Figures and Tables

**Tab. 1:** Compositions of topical sunscreens.

Formul. code	Variables % (w/w)		

	Zinc Oxide	Titanium Dioxide	Ascorbic acid
A	2.5	–	–
B	2.5	–	5
C	5	–	–
D	5	–	1
E	5	–	5
F	–	5	–
G	–	5	5
